# *CDKN2A*-rs10811661 polymorphism, waist-hip ratio, systolic blood pressure, and dyslipidemia are the independent risk factors for prediabetes in a Vietnamese population

**DOI:** 10.1186/s12863-015-0266-0

**Published:** 2015-09-03

**Authors:** Tran Quang Binh, Nguyen Thi Trung Thu, Pham Tran Phuong, Bui Thi Nhung, Trinh Thi Hong Nhung

**Affiliations:** National Institute of Hygiene and Epidemiology, 1 Yersin, Hanoi, 112800 Vietnam; Hanoi National University of Education, 136 Xuan Thuy Street, Hanoi, Vietnam; National Institute of Nutrition, 48B Tang Bat Ho Street, Hanoi, 112807 Vietnam

**Keywords:** Association study, *CDKN2A* gene, Prediabetes, Single nucleotide polymorphism, Vietnamese population

## Abstract

**Background:**

People with prediabetes are at greater risk for heart attack, stroke, kidney disease, vision problems, nerve damage and high blood pressure, compared to those without the disease. Prediabetes is a complex disorder involving both genetic and environmental factors in its pathogenesis. This cross-sectional study aimed to investigate the independent risk factors for prediabetes, considering the contribution of genetic factors (*TCF7L2*-rs7903146*, IRS1-*rs1801278*, INSR-*rs3745551*, CDKN2A*-rs10811661*,* and *FTO-*rs9939609), socio-economic status, and lifestyle factors.

**Results:**

Among the candidate genes studied, the *CDKN2A-*rs10811661 polymorphism was found to be the most significant factor associated with prediabetes in the model unadjusted and adjusted for age, sex, obesity-related traits, systolic blood pressure, dyslipidemia, socio-economic status, and lifestyle factors. In the final model, the *CDKN2A*-rs10811661 polymorphism (OR per T allele = 1.22, 95 % CI = 1.04–1.44, *P* = 0.017), systolic blood pressure (OR per 10 mmHg = 1.14, 95 % CI = 1.08–1.20, *P* < 0.0001), waist-hip ratio (OR = 1.25, 95 % CI = 1.10–1.42, *P* < 0.0001), dyslipidemia (OR = 1.57, 95 % CI = 1.15–2.14, *P* = 0.004), and residence (OR = 1.93, 95 % CI = 2.82–4.14, *P* < 0.0001) were the most significant independent predictors of prediabetes, in which the power of the adjusted prediction model was 0.646.

**Conclusions:**

The study suggested that the *CDKN2A-*rs10811661 polymorphism, waist-hip ratio, systolic blood pressure, and dyslipidemia were significantly associated with the increased risk of prediabetes in a Vietnamese population. The studied genetic variant had a small effect on prediabetes.

**Electronic supplementary material:**

The online version of this article (doi:10.1186/s12863-015-0266-0) contains supplementary material, which is available to authorized users.

## Background

Prediabetes is the condition where blood sugar levels are higher than normal, but not yet high enough to be classified as diabetes [1]. The importance of prediabetes has been underscored by the facts that (i) up to 70 % of people with prediabetes may develop type 2 diabetes (T2D) during their lifetimes [2]; (ii) the average time it takes a person with prediabetes to develop T2D is 3 years [3]; and (iii) people with prediabetes are at greater risk for heart attack, stroke, kidney disease, vision problems, nerve damage and high blood pressure, compared to people without the disease [4, 5]. However, prediabetes is reversible and its related metabolic disorders can be improved with proper treatment [6]. Thus, it is crucial to identify risk factors for prediabetes to prevent a person from developing this disorder.

Predisposition to prediabetes could be determined by many different combinations of genetic variants and environmental factors. Environmental factors that can increase risk for prediabetes and T2D include lifestyle habits (a sedentary lifestyle and poor nutrition, smoking and excessive alcohol consumption), overweight or obese, poor sleep, age, high blood pressure, and abnormal lipid levels [7, 8]. Genetic factors contribute to development of prediabetes and T2D. Defects in genes that encode proteins affect pathways involved in insulin control and glucose homeostasis (the balance of insulin and the hormone glucagon to maintain blood glucose), hence can raise the risk for diabetes. Such genes including *INSR, IRS1, CDKN2A, TCF7L2,* and *FTO* are also identified in genome wide association (GWA) studies [9, 10]. The contributions of these genetic variants on T2D vary among different ethnic populations because of the differences in environmental factors, risk–factor profiles, and genetic background [8, 11]. It is unclear whether these variants have the same effect in Vietnamese population, which has different socio–economic and genetic background. Moreover, the importance of each risk factor for prediabetes which varies within a specific population needs to be clarified. To date, there has been a limited data on risk factors for prediabetes in Vietnamese population. Therefore, the study was designed to investigate both genetic (*TCF7L2*-rs7903146*, IRS1-*rs1801278*, INSR*-rs3745551*, CDKN2A*-rs10811661*,* and *FTO*-rs9939609) and environmental factors for prediabetes in a Vietnamese population. The most significant factors associated with prediabetes were also reported.

## Methods

### Subjects and data collection

The study included 2,610 subjects (411 prediabetic cases and 2,199 normoglycemic controls). They were recruited from a cross-sectional and population-based study to be representatives of prediabetic subjects and normoglycemic controls in the general population of the Red River Delta, Vietnam. Of the total 2,610 participants, 2,608 (99.9 %) belonged to Kinh ethnic group. The Ethics Committee of the National Institute of Hygiene and Epidemiology, Vietnam approved the study. All participants provided written informed consent before entering the study. The details of the survey to collect data were reported previously [12]. In summary, data were collected on social-economic status (current age, gender, ethnicity, educational level, occupation, marital status, income level), lifestyle patterns (residence, alcohol consumption, smoking history, time spent for night’s sleep, siesta, and watching television), family history of diabetes, medical and reproductive history. Anthropometric parameters measured included weight, height, waist circumference (WC), hip circumference (HC), percent body fat, systolic blood pressure (SBP), and diastolic blood pressure (DBP). Blood samples were collected and centrifuged immediately in the morning after a participant had fasted for at least 8 h prior to the clinic visit. Plasma glucose was measured by glucose oxidase method (GOD–PAP). Lipid profile including total cholesterol (TC), triglycerides (TG), high-density lipoprotein cholesterol (HDL-C), and low-density lipoprotein cholesterol (LDL-C) were measured by enzymatic methods. Glucose and lipid profile were analyzed using a semi–autoanalyzer (Screen Master Lab; Hospitex Diagnostics LIHD112, Italy) with commercial kit (Chema. Diagnostica, Italy). Dyslipidemia [13] is defined as HDL-C < 40 mg/dL for men and < 50 mg/dL for women, and TC, LDL-C and TG levels ≥ 200, ≥ 130 and ≥ 130 mg/dL, respectively.

The glycaemic status of subjects was determined using fasting plasma glucose level (FPG) and oral glucose tolerance test (OGTT) with 75 g glucose [14]. Participants were classified as having diabetes if they had FPG ≥ 7.0 mmol/l or 2-h plasma glucose ≥ 11.1 mmol/l or previous diagnosis of diabetes and current use of drug for its treatment. Normal glucose tolerance (NGT) was classified when FPG < 5.6 mmol/l and 2-h plasma glucose < 7.8 mmol/l. Isolated impaired fasting glucose (IFG) was identified if FPG was between 5.6 and 6.9 mmol/l, and 2-h plasma glucose was less than 7.8 mmol/l. Isolated impaired glucose tolerance (IGT) was classified if FPG was less than 5.6 mmol/l and 2-h plasma glucose was between 7.8 and 11.0 mmol/l. Combined IFG and IGT (IFG − IGT) were determined if FPG was between 5.6 and 6.9 mmol/l, and 2-h plasma glucose was between 7.8 and 11.0 mmol/l. Prediabetic status included IFG and/or IGT.

### Genotyping

Peripheral blood samples were obtained from each participant and genomic DNA was extracted from peripheral blood leukocytes, using Wizard® Genomic DNA Purification Kit (Promega Corporation, USA). Primers, protocols of polymerase chain reaction, and restriction enzymes for genotyping the polymorphisms are presented in Additional file 1. Our typing strategy was to use the allele–specific primer (ASP) typing method [15], then 10 % of all samples were typed using restriction fragment length polymorphism (RFLP) analysis to validate observed results. There were more than 98 % agreement of the result between ASP typing method and RFLP analysis in the samples checked. In addition, samples were selected randomly and re-genotyped using the original platform. The results showed that the concordance rate was 96–99 % with respect to the 30 % of samples genotyped twice for quality control.

### Statistical analysis

Genotypes were coded as 0, 1, and 2, depending on the number of copies of risk alleles. Genotype frequencies were compared and tested for Hardy–Weinberg equilibrium (HWE) by Fisher’s exact test. Five genetic models were tested (dominant, co-dominant, over-dominant, recessive, and additive model). Akaike’s Information Criterion and Bayesian Information Criterion were applied to estimate the best-fit model for each SNP. The procedure was performed in SNPstat software [16].

Quantitative variables were checked for normal distribution and compared using Mann–Whitney U test. Binary logistic regression analysis was used to test several models for the associations of prediabetes with the risk alleles and other variables, taken into account the covariates (age, sex, socio-economic status, lifestyle factors, obesity–related traits (BMI, WC, HC, WHR, and percent body fat), systolic blood pressure, and lipid profile). The variables included in the analyses were checked for multicollinearity to ensure the stability of the parameter estimates. Here, data are presented as odds ratios with 95 % confidence intervals (CI). In order to assess the model performance, a receiver operating characteristic (ROC) curve was built to plot probabilities resulted from the multivariate logistic regression analysis, and the area under ROC curve (AUC) was used to measure the power to predict individuals with prediabetes. The level of significance was set to 0.05 for all analyses. The above statistical procedures were performed using SPSS version 16.0 (SPSS, Chicago, USA). The Bayesian model averaging was used to cross-validate the final model using Bayesian Model Averaging Software with the R Statistical Environment version 3.1.3 [17].

## Results

### Characteristics of the study subjects

Of the 2,610 participants recruited into the study, 65.4 % were women, 72.6 % were farmers, and 72.2 % had elementary or intermediate levels of education. The characteristics of subjects in prediabetic cases and controls are shown in Table 1. There were significant differences between prediabetic and control groups in age, BMI, waist circumference, WHR, systolic blood pressure, diastolic blood pressure, total cholesterol, HDL − C, and triglyceride. Significant differences between cases and controls were not found in gender, height, weight, body fat percent, hip circumference, nutrition status, and LDL − C.

**Table 1:** Characteristics of subjects in prediabetic cases and controls

Characteristics	Prediabetic cases	Controls	Total	*P* − value
	(*N* = 411)	(*N* = 2199)	(*N* = 2610)	
Male, *n* (%)	152 (37 %)	752 (34.2 %)	904 (34.6 %)	0.276
Age (year)	53 (47–57.8)	51 (46–56)	51 (46–56)	<0.0001
Weight (kg)	51.8 (46–57.9)	51(46.3 − 56.5)	51 (46.2 − 56.6)	0.117
Height (cm)	155.5 (150.5 − 160)	155 (150.7 − 160)	155 (150.6 − 160	0.731
Body mass index (kg/m^2^)	21.5 (19.6 − 23.4)	21.1 (19.3 − 22.9)	21.2 (19.4 − 23)	0.012
Body fat (%)	28.2 (23.7 − 31.9)	27.5 (22.9 − 31.5)	27.6 (23.2 − 31.6)	0.119
Waist circumference (cm)	75 (69–82)	73.5 (68.5 − 79)	74 (68.5 − 79.5)	0.002
Hip circumference (cm)	88 (84–92)	88 (84–91.3)	88 (84–91.5)	0.628
Waist − hip ratio	0.85 (81–0.90)	0.84(0.80 − 0.88)	0.84 (0.80 − 0.88)	<0.0001
Nutrition status				
Normal	237 (57.8)	1342 (61.4)	1579 (60.8)	0.129
Overweight	77 (18.8)	344 (15.7)	421 (16.2)	
Obesity	44 (10.7)	183 (8.4)	227 (8.7)	
Underweight	52 (12.7)	316 (14.5)	368 (14.2)	
Systolic blood pressure (mmHg)	120 (110–137.5)	110.3 (100–127.5)	115 (110–130)	<0.0001
Diastolic blood pressure (mmHg)	80 (70–85)	70 (65–80)	70 (65–80)	<0.0001
Total cholesterol (mmol l^−1^)	4.60 (4.09 − 5.00)	4.20 (3.85 − 4.87)	4.30 (3.90 − 4.90)	<0.0001
HDL − C (mmol l^−1^)	1.19 (0.97 − 1.60)	1.23 (0.99 − 1.60)	1.22 (0.98 − 1.60)	<0.0001
LDL − C (mmol l^−1^)	3.10 (2.64 − 3.70)	2.79 (2.31 − 3.31)	2.83 (2.34 − 3.40)	0.103
Triglyceride (mmol l^−1^)	1.80 (1.12 − 2.55)	1.34 (1.00 − 2.02)	1.41 (1.01 − 2.10)	<0.0001

HDL − C, high-density lipoprotein − cholesterol; LDL − C, low-density lipoprotein − cholesterol. Quantitative data are median (interquartile range). Qualitative data are number (%). *P*-value by Mann–Whitney U test or chi-square test

### Associated factors for prediabetes

Socioeconomic status (age, marital status), lifestyle patterns (residence, alcohol consumption), anthropometric traits (BMI, WC, WHR, and SBP), and lipid profile (TC, TG, and LDL-C) were significantly associated with prediabetes in univariate logistic regression (Additional file 2). The analysis of the best-fit model for individual SNPs in candidate genes with prediabetes among genetic models of inheritance (additive, codominant, dominant, overdominant, and recessive) is shown in Additional file 3. The lowest values of both Akaike’s Information Criterion and Bayesian Information Criterion were only found in the additive model, indicating this best-fit model in all studied SNPs.

**Fig. 1:**
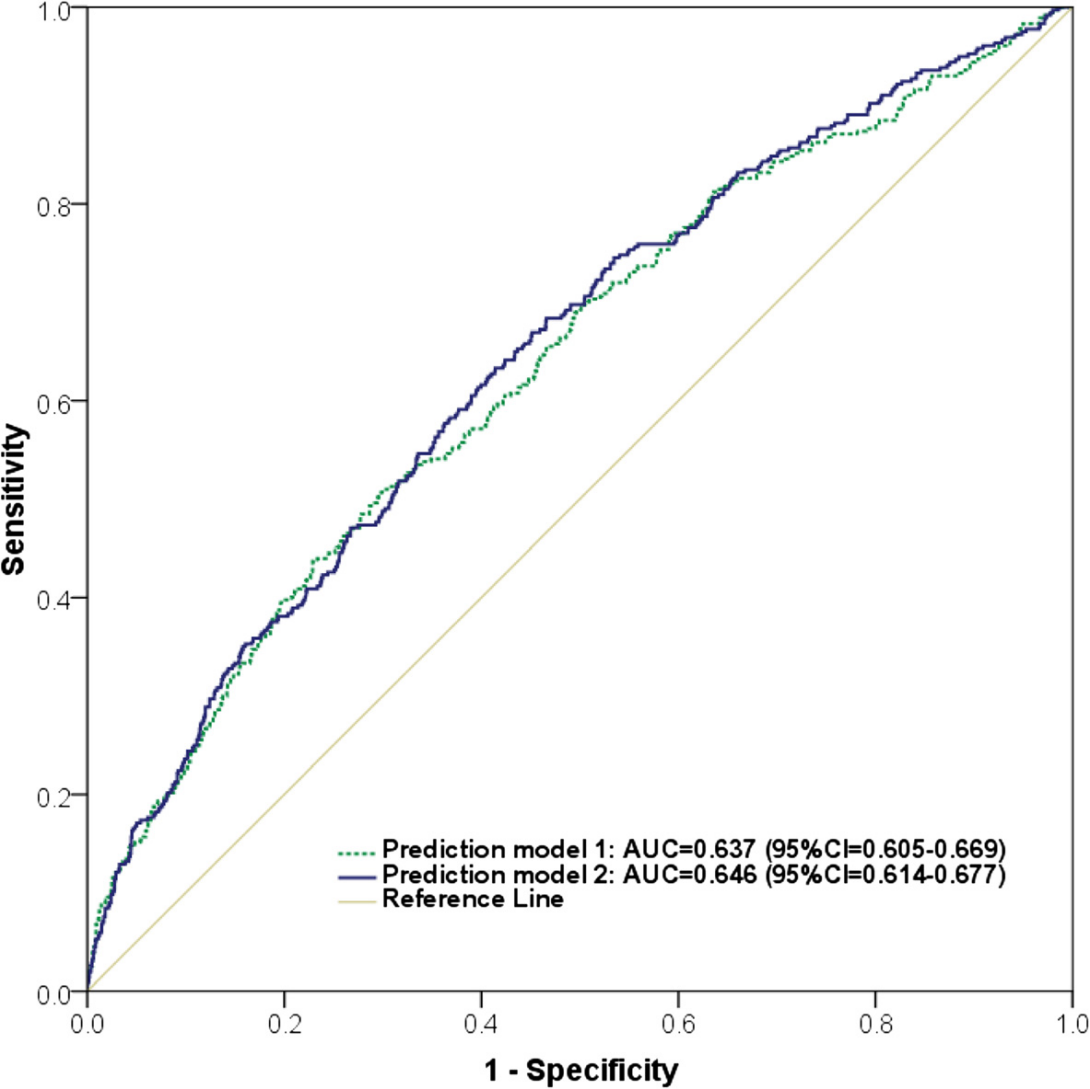
ROC curvers for the prediction models on the number of risk allele of *CDKN2A*-rs10811661, residence, waist-hip ratio, systolic blood pressure, and dyslipidemia in model 2 and model 1 without genetic marker

The association of prediabetes with residence, marital status, alcohol consumption, WHR, SBP, dyslipidemia, and *CDKN2A*-rs10811661 polymorphism was observed in multivariate analysis (Table 2), considering the contribution of genetic factors, anthropometric measurements, lipid profile, socio-economic status and lifestyle factors. The prediction model using the most significant predictors of prediabetes is presented in Table 3. In the final model, the *CDKN2A-*rs10811661 polymorphism (OR per T allele = 1.22, 95 % CI = 1.04–1.44, *P* = 0.017), systolic blood pressure (OR per 10 mmHg = 1.14, 95 % CI = 1.08–1.20, *P* < 0.0001), waist–hip ratio (OR = 1.25, 95 % CI = 1.10–1.42, *P* < 0.0001), dyslipidemia (OR = 1.57, 95 % CI = 1.15–2.14, *P* = 0.004), and residence (OR = 1.93, 95 % CI = 2.82–4.14, *P* < 0.0001) were the most significant independent predictors of prediabetes. The independent variables in the final model were also confirmed using the Bayesian model averaging (Additional file 4). The area under ROC curve for the prediction model of prediabetes on the predictors including residence, waist-hip ratio, and systolic blood pressure, dyslipidemia and *CDKN2A*-rs10811661 polymorphism was 0.646 (95 % CI: 0.614 − 0.677, *P* < 0.0001). Adding the genetic marker to the clinical covariates improved the area under ROC curve slightly from 0.637 to 0.646 (*P* < 0.019, Wilcoxon Signed Ranks Test) (Fig. 1).

**Table 2:** Multivariate analysis of association for prediabetes

Variable	OR (95 % CI)	*P-value*	Variable	OR (95 % CI)	*P-value*
Sex			Residence		
Female	1		Rural	1	
Male	0.78 (0.45–1.33)	0.354	Urban	3.95 (2.50–6.23)	<0.0001
Age (year)	1.02 (0.99–1.04)	0.075	Alcohol consumption		
Marital status			None	1	
Married	1		< 1 drink/mo	1.14 (0.62–2.09)	0.686
Never	2.14 (1.01–4.55)	0.048	≥ 1 drink/mo to < 1 drink/wk	2.02 (1.15–3.56)	0.015
Widowed	0.92 (0.53–1.60)	0.766	1 drink/wk to ≤ 1 drink/d	1.49 (0.87–2.55)	0.147
Others	0.87 (0.29–2.57)	0.800	≥ 2 drink/d	2.06 (1.16–3.68)	0.014
Education level			Smoking		
Elementary	1		None	1	
Intermediate	0.97 (0.63–1.50)	0.904	Current smoker	0.75 (0.44–1.28)	0.293
Secondary	0.99 (0.56–1.76)	0.987	Ex–smoker	1.01 (0.57–1.77)	0.987
Post–secondary	0.89 (0.48–1.62)	0.691	Watching televison time/day		
Heavy occupation			≤ 3 h	1	
Yes	1		> 3 h	0.81 (0.44–1.51)	0.513
No	0.95 (0.64–1.41)	0.800	Sleeping time/day		
Income level			6–7 h	1	
< 25 percentiles	1		< 6 h	0.90 (0.62–1.31)	0.584
25– < 50 percentiles	1.25 (0.86–1.82)	0.246	≥ 8 h	0.99 (0.72–1.39)	0.996
50–75 < percentiles	0.94 (0.63–1.40)	0.749	Sitting time/day		
≥ 75 percentiles	0.99 (0.67–1.48)	0.984	≤ 4 h	1	
			> 4 h	0.96 (0.73–1.28)	0.790
Systolic blood pressure (SD = 10 mmHg)	1.11 (1.04–1.19)	0.001	Siesta time/day (SD = 15 min)	1.08 (1.01–1.15)	0.020
Dyslipidemia			Each of the following obesity-related measurements:		
No	1		Waist-hip ratio (SD = 0.07)	1.21 (1.03–1.41)	0.019
Yes	1.48 (1.04–2.09)	0.027	Waist circumference (SD = 7 cm)	1.12 (0.98–1.27)	0.086
*CDKL2A*-rs10811661 per copy of T allele	1.23 (1.03–1.46)	0.022	Hip circumference (SD = 7 cm)	1.01 (0.85–1.21)	0.873
*TCF7L2*-rs7903146 per copy of T allele	1.13 (0.64–2.01)	0.676	Body mass index (SD = 0.25 kg/m^2^)	1.12 (0.99–1.28)	0.079
*IRS1-*rs1801278 per copy of G allele	1.01 (0.57–1.79)	0.967	Body fat (SD = %)	1.22 (0.90–1.66)	0.206
*INSR-*rs3745551 per copy of G allele	1.05 (0.86–1.27)	0.648			
*FTO-*rs9939609 per copy of A allele	0.98 (0.79–1.22)	0.852			

SD, standard deviation. One drink was defined as a 50–ml cup of rice wine at about 30 %

**Table 3:** The most significant independent predictors of prediabetes

Variable	Unit	Coefficient	Odds ratio (95 % CI)	*P*–value
Waist to hip ratio	0.07	0.224	1.25 (1.10 − 1.42)	<0.0001
Systolic blood pressure	10 mmHg	0.124	1.13 (1.07 − 1.20)	<0.0001
Dyslipidemia	0 = no, 1 = yes	0.453	1.57 (1.15 − 2.14)	0.004
Residence	0 = rural, 1 = urban	1.038	1.93 (2.82 − 4.14)	<0.0001
*CDKN2A-*rs10811661	Number of T allele	0.201	1.22 (1.04 − 1.44)	0.017
Intercept		−6.581	-	<0.0001

*P*-value by multivariate logistic regression

## Discussion

Of the 5 candidate SNPs tested for association, we found that the *CDKN2A*-rs10811661 polymorphism was significantly associated with prediabetes in a Vietnamese population, independent of obesity-related traits, considering the influence of the socio-economic status and lifestyle factors. The association of the *CDKN2A*-rs10811661 polymorphism with T2D was initially reported in White populations [18–20], and subsequently replicated in Asian populations [21–23]. The *CDKN2A*-rs10811661 polymorphism was significantly associated with T2D in Japaneses (OR = 1.25, 95 % CI = 1.08–1.45, *P* = 0.0024) [22], and in Indians (OR = 1.37, 95 % CI = 1.18–1.59, *P* = 5.1E-05) [24]. In Chinese populations, the *CDKN2A*-rs10811661 polymorphism was associated with increased risk in both prediabetes (OR = 1.23, 95 % CI = 1.11–1.36) and T2D (OR = 1.46, 95 % CI = 1.01–2.11) in a case–control study and confirmed in a prospective study that the risk allele of rs10811661 increased the risk of incident T2D by 94 % [25]. Moreover, in GWA studies in Asians, the variant was associated with T2D in East Asians (Han Chinese and Japanese) (OR = 1.23, 95 % CI = 1.18–1.29, *P* = 1.43E-18) [26], and a large multi-center GWA study replicated the association in both East Asians (OR = 1.25, 95 % CI = 1.17–1.32, *P* = 6.3E-13) and South Asians (OR = 1.20, 95 % CI = 1.11–1.31, *P* = 1.4E-05) [27]. These studies showed that the effect of the variant in *CDKN2A* gene seemed slightly higher in T2D compared to prediabetes in the present study (OR = 1.22, 95 % CI = 1.04–1.44, *P* = 0.017). On the other hand, this polymorphism was not associated with prediabetes in German people [28]. Given the multifactorial pattern of prediabetes, the contribution of the *CDKN2A*-rs10811661 polymorphism varies among populations depending on the socio–economic status, lifestyle factors, genetic background, and risk − factor profile of each population [29].

There were many factors influencing the association between the *CDKN2A*-rs10811661 polymorphism and prediabetes, including bias selection of subjects, confounding factors such as socio–economic condition, and lifestyle factors. The bias selection in the study was controlled since the subjects were recruited from the population − based screening survey with a sample size representative of all prediabetic cases and normoglycemic controls in the general population. Moreover, given the multifactorial nature of prediabetes, the association in our study was investigated in several analysis models, which considered the various factors including sex, age, systolic blood pressure, obesity − related traits (BMI, WC, WHR, and body fat percentage), socio–economic patterns (occupation, education level, residence, marital status, income level), and lifestyle factors (smoking, alcohol consumption, leisure time spent sitting, watching TV, and siesta). Thereby, the statistically significant association between the *CDKN2A*-rs10811661 polymorphism and prediabetes was found to be independent of the traditional risk factors.

Regarding the allele and genotype frequencies of the *CDKN2A*-rs10811661 polymorphism, we found that the risk T allele frequency was 57.6 %, and the frequencies of CC, CT, and TT genotypes were 18.9, 46.9, and 34.2 %, respectively in the total sample. The allele and genotype frequencies in our sample were similar to those in Asian populations (Han Chinese: 57 %, Japanese: 52.4 %) and different from those in European (77.8–80.1 %) and African (89–98.2 %) populations based on HapMap data [30].

Being obesity, which is associated with insulin resistance and dysfunction of beta cell, is one of the most important risk factors for the development of prediabetes [31]. Among obesity-related traits, WHR was recognized to be the most significantly associated with prediabetes in our population. In the present study, the association between the *CDKN2A-*rs10811661 polymorphism and prediabetes was consistently significant when adding each of the obesity − related traits in the analysis models including age, gender, systolic blood pressure, socio-economic status and lifestyle factors, indicating the direct effect of the *CDKN2A*-rs10811661 polymorphism on prediabetes, independently of the obesity–related traits.

In terms of predictors of prediabetes, few genetic studies have been reported although the importance of prediabetes has been underscored. The present data showed an increased prediabetes risk with an additive effect of the alleles of *CDKN2A*-rs10811661 (OR per T allele = 1.22, 95 % CI = 1.04–1.44, *P* = 0.017). Our finding supports the association of the *CDKN2A-*rs10811661 polymorphism with prediabetes reported in previous case–control studies in Asian populations [22, 23, 25, 28]. Moreover, the predictive effect of the *CDKN2A-*rs10811661 polymorphism on the incident T2D was also confirmed in a 3.5 year follow-up study [28]. These findings can be explained by the evidences that a reduced insulin release was observed for the *CDKN2A*-rs10811661 T-allele after both oral and intravenous glucose challenges [20] and that the SNP was significantly associated with early-phase insulin release [32]. Among the independent risk factors for prediabetes, WHR, dyslipidemia, and systolic blood pressure demonstrated the strongest effects in our findings, which is in agreement with previous studies [33–35]. Adding the genetic marker to the clinical covariates in our study improved slightly the area under the receiver operating characteristic curve from 0.637 to 0.646 (*P* < 0.019), indicating that the studied variant had a small effect on prediabetes.

Indeed, some advantages could be highlighted in this study. Since this is a large population-based study in the Red River Delta region, Vietnam, the findings of the study will be interpreted for general population of this region in both genetic pattern and risk factor profile. The studied population could be considered as a homogeneous sample of the Kinh ethnic adults aged 40–64 years in a rural province without other ethnic admixtures. Prediabetes including IFG and/or IGT was determined using fasting plasma glucose level and oral glucose tolerance test with 75 g glucose. This method has been widely accepted and frequently referred as the “gold standard” for diagnosis of prediabetes. However, several limitations should be noted in this study. First, the limitation of the cross-sectional study design does not allow for conclusions of the causal relationships. Next, among many candidate SNPs have been proposed to be associated with T2D and prediabetes, the present study was only interested in 5 SNPs in genes related to insulin pathway, and thereby the studied genetic variant had a small effect on prediabetes despite statistical significance. Lastly, the area under ROC curve of 0.646 shows the poor power of prediction of the model.

## Conclusions

These data demonstrate that the *CDKN2A*-rs10811661 polymorphism, waist–hip ratio, systolic blood pressure, and dyslipidemia were significantly associated with the increased risk of prediabetes in a Vietnamese population. The association remains consistent after adjustment for age, gender, socio-economic status, and lifestyle-related factors. Because of the small contribution of the single *CDKN2A*–rs10811661 polymorphism, it is necessary to conduct a large-scale prospective study on prediabetes and T2D in Vietnamese population.

## Additional files

Additional file 1: Table S1. Methods for genotyping *CDKN2A, FTO, INSR, IRS1, and TCF7L2* polymorphisms. (DOCX 37 kb) Additional file 2: Table S2. Associated factors of prediabetes in middle-aged population in univariate logistic regression analysis. (DOCX 39 kb) Additional file 3: Table S3. Analysis of the best-fit model for individual SNPs of candidate genes for prediabetes. (DOCX 23 kb) Additional file 4: Figure S1. Analysis Bayesian Model Averaging analysis to cross-validate the final model. (DOCX 67 kb)

## Abbreviations

BMI: Body mass index
CI: Confidence interval
DBP: Diastolic blood pressure
HC: Hip circumference
HDL-C: High-density lipoprotein cholesterol
IGT: Impaired glucose tolerance
IFG: Impaired fasting glucose
LDL-C: Low-density lipoprotein cholesterol
OGTT: Oral glucose tolerance test
OR: Odds ratio
RFLP: Restriction fragment length polymorphism
SBP: Systolic blood pressure
TC: Total cholesterol
T2D: Type 2 diabetes
TG: Triglycerides
WC: Waist circumference
WHR: Waist–hip ratio
