# Supplementary material for: CDKN2A-rs10811661 polymorphism, waist-hip ratio, systolic blood pressure, and dyslipidemia are the independent risk factors for prediabetes in a Vietnamese population
Source: BMC Genet. 2015 Sep 3;16:107. doi: 10.1186/s12863-015-0266-0 (PMC4559161; doi:10.1186/s12863-015-0266-0)
Supplement: Additional file 1: Table S1. — Methods for genotyping CDKN2A, FTO, INSR, IRS1, and TCF7L2 polymorphisms. (DOCX 37 kb) [file 12863_2015_266_MOESM1_ESM.docx]

**Table S1. Methods for genotyping *CDKN2A, FTO, INSR, IRS1, and TCF7L2* polymorphisms**

| SNP ID Nearest gene | dbSNP^a^ | Primers | Tm | Restriction enzyme | Allele size (bp) |
| --- | --- | --- | --- | --- | --- |
| *rs10811661*  *CDKN2A* | C>T | 5’- accttcagccacctctctgtctttc-3’  5’- cccatcctgggtaggaggagcc-3’ | 62^o^C | *PagI* | C: 350 bp  T: 290bp + 60bp |
|  | C allele | 5’-tcagttaagcagatgaaattc-3’  5’-ggtaatagacttactgtcatcg-3’ | 53^o^C | *-* | 207 bp |
|  | T allele | 5’-tcagttaagcagatgaaattc-3’  5’-ggtaatagacttactgtcatca-3’ | 53^o^C | *-* | 207 bp |
| *rs9939609*  *FTO* | T>A | 5’-ggctcttgaatgaaatagga-3’  5’-agagactatccaagtgcagtac-3’ | 54^o^C | *ScaI* | T: 170 bp  A:150bp + 20bp |
|  | A allele | 5’-ggctcttgaatgaaatagga-3’  5’-agactatccaagtgcatcaga-3’ | 62^o^C | *-* | 168 bp |
|  | T allele | 5’-ggctcttgaatgaaatagga-3’  5’-agactatccaagtgcatcagt-3’ | 62^o^C | *-* | 168 bp |
| *rs3745551*  *INSR* | A/G | 5’-ctggctggtaatattcttctc-3’  5’-gaacaggtgggaatgctag-3’ | 52^o^C | *NheI* | A: 213bp  G:194bp + 18bp |
|  | A allele | 5’-ctggctggtaatattcttctc-3’  5’-gaacaggtgggaatgcttat-3’ | 55^o^C | *-* | 213bp |
|  | G allele | 5’-ctggctggtaatattcttctc-3’  5’-gaacaggtgggaatgcttcc-3’ | 55^o^C | *-* | 213 bp |
| *rs1801278*  *IRS1* | A>G | 5’-ctttccacagctcaccttc-3’  5’-tgtttcgcatgtcagcatag -3’ | 54^o^C | *Eco88I (AvaI)* | A:196bp+115bp  G:168bp+115bp+28 bp |
|  | A allele | 5’-ctttccacagctcaccttc-3’  5’-gtaggcctgcaaatgctagcagccaa-3’ | 57^o^C | *-* | 190bp |
|  | G allele | 5’-ctttccacagctcaccttc-3’  5’-gtaggcctgcaaatgctagcagccag-3’ | 57^o^C | *-* | 190bp |
| *rs7903146*  *TCF7L2* | T>C | 5’-acaattagagagctaagcactttttaggta-3’  5’-gtgaagtgcccaagcttctc-3’ | 62^o^C | *FastDigest RsaI* | T: 188bp  C:159bp + 29bp |
|  | C allele | 5’-agagctaagcactttttagattc-3’  5’-gtgaagtgcccaagcttctc-3’ | 53^o^C | *-* | 165bp |
|  | T allele | 5’-agagctaagcactttttagattt-3’  5’-gtgaagtgcccaagcttctc-3’ | 53^o^C | *-* | 165bp |

^a^ Accession number of each polymorphism to dbSNP at <http://www.ncbi.nlm.nih.gov/>.
