# Supplementary material for: CDKN2A-rs10811661 polymorphism, waist-hip ratio, systolic blood pressure, and dyslipidemia are the independent risk factors for prediabetes in a Vietnamese population
Source: BMC Genet. 2015 Sep 3;16:107. doi: 10.1186/s12863-015-0266-0 (PMC4559161; doi:10.1186/s12863-015-0266-0)
Supplement: Additional file 2: Table S2. — Associated factors of prediabetes in middle-aged population in univariate logistic regression analysis. (DOCX 39 kb) [file 12863_2015_266_MOESM2_ESM.docx]

**Table S 2. Associated factors of prediabetes in middle-aged population in univariate logistic regression analysis**

| **Social-economic status** | **OR (95%CI)** | ***P*-value** | **.** | **Lifestyle patterns** | **OR (95%CI)** | ***P*-value** |
| --- | --- | --- | --- | --- | --- | --- |
| Sex |  |  |  | Residence |  |  |
| Female | 1 |  |  | Rural | 1 |  |
| Male | 1.13 (0.91−1.41) | 0.276 |  | Urban | 2.99 (2.08−4.32) | < 0.0001 |
| Age (year) | 1.04 (1.02−1.05) | <0.0001 |  | Alcohol consumption |  |  |
| Marrital status |  |  |  | None | 1 |  |
| Married | 1 |  |  | <1 drink/mo | 1.06 (0.68−1.66) | 0.793 |
| Never | 2.27 (1.20−4.26) | 0.011 |  | ≥ 1 drink/mo to < 1 drink/wk | 1.60 (1.06−2.40) | 0.025 |
| Widowed | 1.22 (0.79−1.90) | 0.369 |  | 1 drink/wk to ≤ 1 drink/d | 1.29 (0.95−1.75) | 0.104 |
| Others | 0.81 (0.31−2.08) | 0.661 |  | ≥ 2 drink/d | 1.37 (0.98−1.93) | 0.066 |
| Education level |  |  |  | Smoking |  |  |
| Elementary | 1 |  |  | None | 1 |  |
| Intermediate | 0.98 (0.68−1.39) | 0.895 |  | Current smoker | 0.97 (0.72−1.30) | 0.832 |
| Secondary | 0.93 (0.60−1.46) | 0.764 |  | Ex–smoker | 1.35 (0.97−1.88) | 0.080 |
| Post–secondary | 1.02 (0.66−1.56) | 0.944 |  | Watching TV time/day |  |  |
| Heavy occupation |  |  |  | ≤ 3 hours | 1 |  |
| Yes | 1 |  |  | > 3 hours | 1.02 (0.63−1.67) | 0.937 |
| No | 1.18 (0.92−1.53) | 0.196 |  | Sleeping time/day |  |  |
| Income level |  |  |  | 6–7 hours | 1 |  |
| < 25 percentiles | 1 |  |  | < 6 hours | 1.12 (0.83−1.52) | 0.466 |
| 25–<50 percentiles | 1.25 (0.93−1.68) | 0.138 |  | ≥ 8 hours | 0.99 (0.77−1.29) | 0.991 |
| 50–75< percentiles | 1.06 (0.77−1.44) | 0.730 |  | Sitting time/day |  |  |
| ≥75 percentiles | 1.24 (0.92−1.68) | 0.158 |  | ≤ 4 hours | 1 |  |
| **Anthropometric measurements** | **OR (95%CI)** | ***P*-value** |  | > 4 hours | 0.97 (0.78−1.22) | 0.818 |
| BMI (kg/m^2^) | 1.05 (1.01−1.09) | 0.016 |  | Siesta time/day | 1.05 (0.99−1.11) | 0.066 |
| Body fat (%) | 1.02 (0.99−1.03) | 0.064 |  | **Serum lipid patterns** | **OR (95%CI)** | ***P*-value** |
| Waist circumference (cm) | 1.02 (1.01−1.04) | 0.001 |  | Dyslilidemia |  |  |
| Hip circumference (cm) | 0.99 (0.98−1.01) | 0.935 |  | No | 1 |  |
| Waist-hip ratio (SD=0.07) | 1.33 (1.18−1.49) | < 0.0001 |  | Yes | 1.62 (1.21−2.17) | 0.001 |
| Systolic blood pressure (SD=10 mmHg) | 1.17 (1.11−1.23) | < 0.0001 |  | High triglycerides |  |  |
| Elevated blood pressure |  |  |  | No | 1 |  |
| Normal | 1 |  |  | Yes | 2.10 (1.68−2.62) | <0.0001 |
| High | 1.91 (1.53−2.38) | < 0.0001 |  | Low HDL-C |  |  |
| Nutrition status |  |  |  | No | 1 |  |
| Normal | 1 |  |  | Yes | 1.21 (0.97−1.51) | 0.094 |
| Overweight | 1.27 (0.96−1.68) | 0.101 |  | High LDL-C |  |  |
| Obesity | 1.36 (0.95−1.95) | 0.090 |  | No | 1 |  |
| Underweight | 0.93 (0.67−1.29) | 0.669 |  | Yes | 2.25 (1.78−2.84) | <0.0001 |
| Abdominal obesity |  |  |  | High total cholesterol |  |  |
| No | 1 |  |  | No | 1 |  |
| Yes | 0.99 (0.73−1.34) | 0.952 |  | Yes | 1.24 (0.93−1.66) | 0.151 |

HDL-C, high-density lipoprotein cholesterol; LDL-C, low-density lipoprotein cholesterol. Abdominal obesity was recorded if waist-hip ratio ≥ 0.90 in males or ≥ 0.80 in females. Elevated blood pressure was defined as systolic blood pressure ≥ 130 mmHg and/or a diastolic blood pressure ≥ 85 mmHg. One drink was defined as a 50–ml cup of rice wine at about 30%.
