# Supplementary material for: CDKN2A-rs10811661 polymorphism, waist-hip ratio, systolic blood pressure, and dyslipidemia are the independent risk factors for prediabetes in a Vietnamese population
Source: BMC Genet. 2015 Sep 3;16:107. doi: 10.1186/s12863-015-0266-0 (PMC4559161; doi:10.1186/s12863-015-0266-0)
Supplement: Additional file 3: Table S3. — Analysis of the best-fit model for individual SNPs of candidate genes for prediabetes. (DOCX 23 kb) [file 12863_2015_266_MOESM3_ESM.docx]

**Table S3 Analysis of the best-fit model for individual SNPs of candidate genes for prediabetes**

| SNP ID Nearest gene | Genetic models | Odds ratio (95% CI ) | *P-*value | AIC | BIC |
| --- | --- | --- | --- | --- | --- |
| *rs10811661*  *CDKN2A* | Additive (T allele) | 1.18 (1.02 – 1.38) | 0.029 | 2223.2 | 2234.9 |
| *C>T* | Codominant |  | 0.088 | 2225.2 | 2242.7 |
|  | CC | 1.0 | - |  |  |
|  | CT | 1.20 (0.88–1.63) | 0.249 |  |  |
|  | TT | 1.41 (1.03–1.93) | 0.034 |  |  |
|  | Dominant |  | 0.089 | 2225.1 | 2236.8 |
|  | CC | 1.0 |  |  |  |
|  | CT+TT | 1.28 (0.96 – 1.71) |  |  |  |
|  | Overdominant |  | 0.660 | 2227.8 | 2239.5 |
|  | CC+TT | 1.0 |  |  |  |
|  | CT | 0.95 (0.77 – 1.18) |  |  |  |
|  | Recessive |  | 0.062 | 2224.4 | 2236.1 |
|  | CC+CT | 1.0 |  |  |  |
|  | TT | 1.23 (0.99 – 1.54) |  |  |  |
| *rs9939609*  *FTO* | Additive (A allele) | 0.92 (0.76 – 1.12) | 0.391 | 2258.3 | 2270.1 |
| T>A | Codominant |  | 0.677 | 2260.3 | 2277.9 |
|  | TT | 1.0 | - |  |  |
|  | TA | 0.93 (0.74–1.18) | 0.570 |  |  |
|  | AA | 0.80 (0.44–1.45) | 0.461 |  |  |
|  | Dominant |  | 0.461 | 2258.5 | 2270.3 |
|  | TT | 1.0 |  |  |  |
|  | AT+AA | 0.92 (0.73 – 1.15) |  |  |  |
|  | Overdominant |  | 0.632 | 2258.9 | 2270.6 |
|  | AA+TT | 1.0 |  |  |  |
|  | AT | 0.94 (0.75 – 1.19) |  |  |  |
|  | Recessive |  | 0.499 | 2258.7 | 2270.4 |
|  | AT+TT | 1.0 |  |  |  |
|  | AA | 0.82 (0.45 – 1.48) |  |  |  |
| *rs3745551*  *INSR* | Additive (G allele) | 0.97 (0.81 – 1.16) | 0.722 | 2051.9 | 2063.5 |
| A>G | Codominant |  | 0.760 | 2053.5 | 2070.8 |
|  | AA | 1.0 | - |  |  |
|  | AG | 1.02 (0.81–1.28) | 0.892 |  |  |
|  | GG | 0.85 (0.53–1.37) | 0.504 |  |  |
|  | Dominant |  | 0.932 | 2052.1 | 2063.6 |
|  | AA | 1.0 |  |  |  |
|  | AG+GG | 0.99 (0.79 – 1.24) |  |  |  |
|  | Overdominant |  | 0.778 | 2052 | 2063.5 |
|  | AA+GG | 1.0 |  |  |  |
|  | AG | 1.03 (0.83 – 1.30) |  |  |  |
|  | Recessive |  | 0.478 | 2051.5 | 2063.1 |
|  | AA+AG | 1.0 |  |  |  |
|  | GG | 0.85 (0.53 – 1.35) |  |  |  |
| *rs1801278*  *IRS1* | Additive (G allele) | 1.08 (0.65 – 1.80) | 0.760 | 2154.3 | 2165.9 |
|  | Overdominant |  | 0.985 | 2154.4 | 2166 |
| A>G | AA+GG | 1.0 |  |  |  |
|  | AG | 1.01 (0.59 – 1.71) |  |  |  |
|  | Recessive |  | 0.879 | 2154.3 | 2165.9 |
|  | AA+AG | 1.0 |  |  |  |
|  | GG | 1.04 (0.61 – 1.77) |  |  |  |
| *rs7903146*  *TCF7L2* | Additive | 1.29 (0.81 – 2.04) | 0.280 | 2257.7 | 2269.4 |
|  | Codominant |  | 0.341 | 2259.7 | 2277.3 |
|  | CC | 1.0 | - |  |  |
|  | CT | 1.28 (0.78–2.10) | 0.336 |  |  |
|  | TT | 1.81 (0.19–17.4) | 0.608 |  |  |
|  | Dominant |  | 0.299 | 2257.8 | 2269.5 |
|  | CC | 1.0 |  |  |  |
|  | CT+TT | 1.30 (0.80 – 2.11) |  |  |  |
|  | Overdominant |  | 0.339 | 2257.9 | 2269.7 |
|  | CC+TT | 1.0 |  |  |  |
|  | CT | 1.28 (0.78 – 2.10) |  |  |  |

SNP, Single Nucleotide Polymorphism; AIC, Akaike’s Information Criterion; BIC, Bayesian Information Criterion
