# Supplementary material for: CDKN2A-rs10811661 polymorphism, waist-hip ratio, systolic blood pressure, and dyslipidemia are the independent risk factors for prediabetes in a Vietnamese population
Source: BMC Genet. 2015 Sep 3;16:107. doi: 10.1186/s12863-015-0266-0 (PMC4559161; doi:10.1186/s12863-015-0266-0)
Supplement: Additional file 4: Figure S1. — Analysis Bayesian Model Averaging analysis to cross-validate the final model. (DOCX 67 kb) [file 12863_2015_266_MOESM4_ESM.docx]

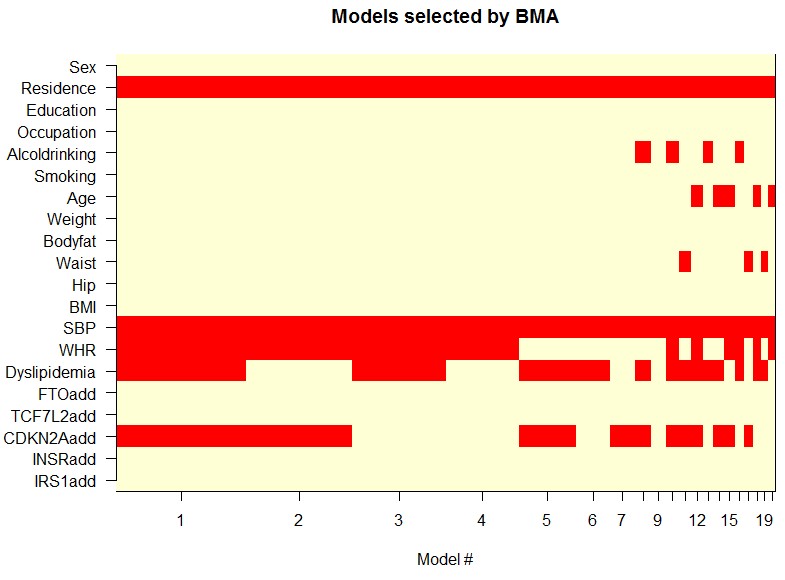


**Figure S1 Analysis Bayesian Model Averaging analysis to cross-validate the final model**

The above figure shows the finding of the Bayesian Model Averaging (BMA) analysis to cross-validate the final model found by multivariate logistic regression analysis.

There were 20 models selected by BMA, in which the best model was model 1 including variables: residence, SBP (systolic blood pressure), WHR (waist–hip ratio), dyslipidemia, and *CDKN2A-*rs10811661(CDKN2Aadd). These variables were presented in most of the selected models.

**Abbreviations:**

Alcoldrinking, alcohol consumption; BMI, body mass index; SBP, systolic blood pressure; Waist, waist circumference; Hip, hip circumference; WHR, waist–hip ratio; FTOadd, *FTO-*rs9939609; TCF7L2add, *TCF7L2-*rs7903146; CDKN2Aadd, *CDKN2A-*rs10811661; INSRadd, *INSR-*rs3745551; IRS1add, *IRS1-*rs1801278.
